# Traumatic pseudoaneurysm following ankle trauma

**DOI:** 10.1259/bjrcr.20150132

**Published:** 2015-07-27

**Authors:** M C A M Melenhorst, K van der Mooren, R C van Nieuwenhuizen, A F J Wüst

**Affiliations:** ^1^ Departement of Radiology, VU Medical Centre, Amsterdam, The Netherlands; ^2^ Department of Radiology, St. Lucas Andreas Hospital, Amsterdam, The Netherlands; ^3^ Department of Vascular Surgery, St. Lucas Andreas Hospital, Amsterdam, The Netherlands

## Abstract

False aneurysms following inversion trauma of the ankle are very uncommon. We present a case of a 40-year-old male referred to our radiology department with persisting and painful swelling of the ankle following an inversion trauma. An MRI scan was performed that showed a false aneurysm originating from a distal anterior tibial artery side branch; the lateral malleolar artery. The false aneurysm was confirmed with ultrasound and successfully treated with ultrasoundguided thrombin injection. The patient made an uneventful recovery.

## Summary

False aneurysms following inversion trauma of the ankle are rare. We report a case of a 40-year-old male referred to our radiology department with persistent swelling of the ankle as a consequence of an inversion trauma. An MRI scan was performed that showed a false aneurysm originating at the lateral malleolar artery, which is a side branch of the anterior tibial artery. The aneurysm was confirmed with an ultrasound and successfully treated with an ultrasound-guided thrombin injection.

## Background

Traumatic pseudoaneurysm of the anterior tibial artery is a rare entity with only a few cases reported in the literature. In these cases, aneurysms were often a complication of ankle arthroscopy and rarely occured following ankle distortion injury. Surgery, ultrasound-guided compression therapy and coil embolization treatment for pseudoaneurysms have been reported before.^1–4^

We report the clinical and radiological findings of an anterior tibial artery pseudoaneurysm caused by an ankle distortion injury that was successfully treated by a thrombin injection.

## Clinical Presentation

A 40-year-old male presented to our orthopaedics clinic with persistent pain and swelling secondary to an inversion injury of the ankle 4 weeks prior to presentation. On physical examination, swelling of the lateral malleolus was noted. Function was limited with limitation in range of motion and inability to put weight on the ankle; therefore, the patient was using crutches. Stability seemed normal. A pulsatile swelling or ecchymosis was not objectified. The patient was not using any kind of antiplatelet or anticoagulant medication. An X-ray of the ankle was unremarkable. Because of the patient's sustained complaints, an MRI scan was performed shortly after the orthopaedic consult to evaluate the swelling, the ankle joint and ligaments.

**Figure 1.**
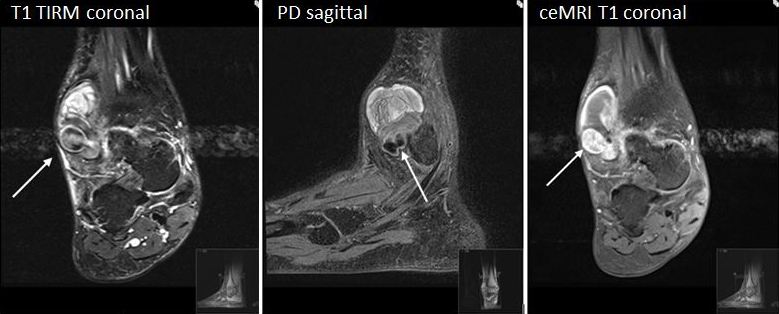
(a) *T*_1_ TIRM coronal view. Large haematoma with cavern causing pulsation artefact (arrow). (b) PD sagittal view. Haematoma around the lateral malleolus with a false aneurysm (arrow). (c) *T*_1_ fat suppression with contrast (ceMRI). Coronal view. Strong enhancement of the false aneurysm (arrow). ceMRI, contrast-enhanced MRI; PD, proton density; TIRM, turbo inversion recovery magnitude sequence.

**Figure 2.**
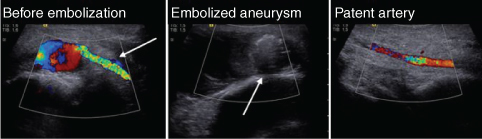
(a) Ultrasound Doppler image. Side branch of the anterior tibial artery (arrow) with connecting false aneurysm. (b) Ultrasound image of the aneurysm after thrombin injection showing no flow (arrow). (c) Ultrasound image showing normal flow in the anterior artery after thrombin injection.

The MRI showed an inhomogeneous mass alongside the lateral malleolus with a maximal diameter of 45 mm. A pulsation artefact originating from a cavern within the mass was present caudally (Figure 1a,b). Contrast series with Dotarem® (gadoterate meglumine, Guerbet, France; 0.5μmol ml^−1^, administered dose 15 ml) showed enhancement of the cavern and a false aneurysm was suspected (Figure 1c). Ultrasound investigation showed a haematoma with a pulsating cavern, which confirmed the diagnosis of a false aneurysm. To-and-fro Doppler signal was demonstrated within the cavern (yin and yang sign, Figure 2a). A connection with the lateral malleolar artery, a side branch of the distal anterior tibial artery, was identified. The aneurysm had a small neck and therefore immediate treatment was possible. The aneurysm was successfully embolized by ultrasound-guided injection of approximately 1 ml 500IE thrombin per ml in calcium chloride 40μmol ml^−1^ (Tissucol® Baxter, USA) using a 21-gauge needle (Figure 2b). Patency of the anterior tibial artery after treatment was established (Figure 2c) and during the following week, the swelling and pain diminished. 1 week later, flow in the anterior tibial artery remained normal, with no flow in the aneurysm. The patient made an uneventful recovery and was discharged from follow-up 6 weeks after the embolization.

## Discussion

A pseudoaneurysm is a false aneurysm of an artery, most often post traumatic in origin. Unlike a true aneurysm, a false aneurysm does not consist of the three normal arterial layers. However, communication with the artery persists. Pseudoaneurysms can easily be detected with an ultrasound or an MRI.^5^

Even though some aneurysms thrombose spontaneously, treatment is often needed owing to the risk of enlargement and, eventually, rupture.^6^

False aneurysms of the anterior tibial artery are extremely rare and can be divided into two categories; aneurysms following surgery and aneuryms following trauma.

A surgical cause can be arthroscopy. Jang et al^3^ describes a case of a pseudoaneurysm following arthroscopy that was successfully treated with ultrasound compression. Another surgical case is described by Inamdar et al.^5^ They report a case of a false aneurysm of the proximal anterior tibial artery following tibial nailing that was surgically resected.

A traumatic cause can be ankle distortion, such as in our case, or ankle fracture. In literature, Ramdass^1^ decribes a case of a false aneurysm of the peroneal artery following ankle distortion. Of interest in this case is the anatomical variant in which the peroneal artery crossed the ankle joint towards the lateral malleolus. This false aneurysm was also surgically resected.

As reported above, there are several options for treatment by either surgical or radiological intervention. A less invasive treatment option not mentioned yet is coil embolization or endovascular covered stent placement, as described by Craxford et al^2^ and Sadat et al.^4^

However, there is an even less invasive option of treating a pseudoaneurysm, which is ultrasound compression or ultrasound-guided thrombin injection. Both options are quick and minimally invasive alternative therapies. Sometimes, ultrasound compression is inadequate, which leaves an easy and effective alternative: the injection of thrombin under ultrasound guidance.

Thrombin is a potent enzyme that transforms fibrinogen to fibrin, the fundamental component of blood coagulation. Two variants of thrombin are available, human and bovine; bovine thrombin carries a small risk of inducing an allergic reaction as it is a foreign substance.^7^

The advantages of using ultrasound-guided thrombin injection include minimal discomfort to the patient, high efficacy (96–100%), lack of influence of concurrent anticoagulation and rapidity of the procedure.^8^ Procedure-related risks of thrombin injection include arterial thrombosis or distal embolism. However, complications are rare (2%).^7,8^

The injection of thrombin in a pseudoaneurysm following femoral artery puncture is an effective treatment option and therefore well accepted.^9^ Even though thrombin injection in pseudoaneurysms originating from crural arteries have not been reported before, we think it can be an efficient and safe minimally invasive treatment option.

## Learning points

1. Traumatic false aneurysm of the distal anterior tibial artery is a rare complication of ankle distortion that can be detected by MRI and ultrasound.
2. Ultrasound-guided injection with thrombin is a good option in the treatment of traumatic false aneurysms following ankle distortion.
